# The Impact of Cosmetic Surgery on Married Women’s Marital Satisfaction and Self-Concept

**Published:** 2018-05

**Authors:** Nazanin Rita Davai, Kamran Ganji, Abdoljalil Kalantar-Hormozi, Ali Abbaszadeh-Kasbi

**Affiliations:** 1 Department of Clinical Psychology, Islamic Azad University, Science and Research Branch, Tehran, Iran;; 2 Department of Psychology, Malayer Branch, Islamic Azad University, Malayer, Iran;; 3 Department of Plastic and Craniofacial Surgery, Medical College of Shahid Beheshti University of Medical Science, 15 Khordad Hospital, Tehran, Iran;; 4 School of Medicine, Tehran university of Medical Sciences, Tehran, Iran

**Keywords:** Marital satisfaction, Self-concept, Married women, Cosmetic surgery

## Abstract

**BACKGROUND:**

This study aimed to identify the impact of cosmetic surgery on married women’s marital satisfaction and self-concept in Tehran.

**METHODS:**

This study was causal-comparative research. The study population consisted of all women having or applying for cosmetic surgery in Tehran over the second half of 2015. Convenient random sampling was used to select the participants. Enriching and Nurturing Relationship Issues Communication and Happiness (ENRICH) marital satisfaction questionnaire and Rogers’ Self-concept Inventory were used to collect data.

**RESULTS:**

The study sample included 44 individuals having facial cosmetic procedures, 51 individuals applying for cosmetic surgery and 55 non-applicants (ordinary people). There were significant differences in terms of marital satisfaction and its components between the cosmetic surgery applicants and surgery group and control group. The cosmetic surgery applicants revealed higher levels of satisfaction in comparison with the surgery and control groups regarding the components’ personality, conflict and leisure. Considering the component marital relationship, the applicant group had higher satisfaction than control group. With regard to the components financial management and relatives and friends, the applicant group had higher satisfaction, in comparison to the control group. The control group had better self-concept than the surgery and applicant groups.

**CONCLUSION:**

Expectation for postoperative positive outcome is an important factor affecting high level of marital satisfaction in surgery applicants. Furthermore, the component self-concept is also a significant predictor of having or applying for cosmetic surgery.

## INTRODUCTION

Today, cosmetic surgery is considered as one of the most common procedures at a global level and its use is increasing.^1^ In 2005, about 10.2 million people underwent cosmetic surgery while, in 2007, approximately, 11.7 million people underwent cosmetic surgery in the USA.^2^^,^^3^ There has been about 70 percent increase in demand for cosmetic surgery since 1990s and its prevalence is growing.^4^ Hawkins defines marital satisfaction as objective feeling of happiness, satisfaction and joy experienced by the couples, when considering all aspects of marriage. Marital satisfaction is an attitudinal variable; therefore, it is regarded as couple’s personal attitudes.^4^

One of the variables affecting the relationship between cosmetic surgery and marital satisfaction is physical attractiveness. Brown *et al.* in their study identified the factors predicting the likelihood of demand for unnecessary (non-clinical) cosmetic surgery and showed that the lower levels of physical attractiveness (self-evaluation) predict the high demand for cosmetic surgery; therefore, it seems that one of the factors that the married cosmetic surgery applicants follow is enhancing physical attractiveness (this probably justifies the demand for women cosmetic surgeries including breast surgery and the like) and promoting marital satisfaction.^5^

Those who feel that they are not physically attractive are more likely to undergo cosmetic surgeries. This idea supports the idea that failure to achieve the social ideals of attractiveness results in higher levels of physical dissatisfaction and finally leads to cosmetic surgeries. A significant point to be noted is that higher levels of physical satisfaction will not be achieved by these people after the surgery if these demands are due to these individuals’ low self-estimate. This reveals the critical role of another variable mediating the impact of cosmetic surgery on expected consequence, i.e. self-concept.^5^

Self-concept is a dynamic system of beliefs, values, sentiments, talents and capabilities. Self-concept refers to one’s self-assessment. This is an individual’s subjective assessment of its own characteristics which may be positive or negative. Positive self-concept indicates that the person accept himself as a person having strengths and weaknesses, leading to his enhance self-confidence in social relations. Negative self-concept reflects the feelings of worthlessness, incompetence and disability.^6^ Self-concept is an overall perception of what we are.^7^

Self-concept is defined as one’s image of himself including actual experiences and the interpretation of these experiences. In general, self-concept is a multi-dimensional and multi-level concept. Body image and self-esteem are studied as the most vital aspects of self-concept.^8^ Lots of studies have dealt with the relationship between body image and self-concept and psychological variables. Some cases revealed the relationship between body image and societal perfectionism and internalization of social messages,^9^ the need to hide perceived flaws from others,^10^ perfectionism^11^ and psychological damages.^12^

The results also indicated the relationship between self-concept and social functioning,^13^ individual goal orientation^14^ and the demand for cosmetic surgery.^15^ According to previous studies, no study has been carried out in Iran researching the impact of cosmetic surgery on marital satisfaction and self-concept as probable factors affecting the demand for cosmetic surgery. Investigating this issue is necessary to determine clinical features of this specific population in order to prevent unnecessary surgery and postoperative adverse outcomes.

## MATERIALS AND METHODS

This study was causal-comparative. The study population consisted of all women having or applying for cosmetic surgery in Tehran over the second half of 2015. Convenient random sampling was used to select the participants. Inclusion criteria were consent and cooperation, surgery date specified by cosmetic surgeon after passing medical procedures and having no facial cosmetic surgery and medical and urgent reasons to perform cosmetic surgery for applicants. Exclusion criteria also contained having no diagnosable mental disorder as well as having cosmetic surgery on one body organ for the second time. 

None of applicants were also selected from the volunteers’ relatives. Since the number of population was not determined, the sample size was decided based on articles published in this field of research. The research sample consisted of 44 individuals having facial cosmetic procedures, 51 individuals applying for cosmetic surgery and 55 non-applicants (ordinary people). Enriching and Nurturing Relationship Issues Communication and Happiness (ENRICH) Marital Satisfaction Questionnaire was used for all participants.

The scale was developed by Olson *et al.* (1989) to assess potentially problematic areas and areas of strengths and in marital relations. ENRICH Questionnaire contains 12 subscales as follows: Idealistic distortion, marital satisfaction, personality issues, communication, conflict resolution, financial management, leisure activities, sexual affairs, children, parenting, family and friends, egalitarianism roles and religious orientation. This questionnaire is composed of strong psychometric properties.^16^ Olson and Olson^17^ estimated the internal consistency of this questionnaire to be 0.73 to 0.90.

The Self-concept Inventory has been used to determine individuals’ positive and negative self-concept and the time required to answer this test is approximately 20 minutes. Rogers’ Self-concept Inventory is an objective test and consists of a 7-score level assigned between two features and one is supposed to select one of the numbers between the two features. This test has two forms A and B. During the presentation of Form A, the participants were asked to mark a number based on the given scale (ranging from one to seven based on closeness to the supposed feature). Then, the participants were asked to specify their ideal personal traits in the Form B.^15^

The calculated scores between 0-7 and 7 and above indicate normal and negative/weak self-concept, respectively. In other words, high scores obtained for this scale show a mismatch between the actual self and the ideal self. Pasha *et al.*^15^ in their study reported the reliability of this scale using split-half method and Cronbach’s alpha to be 0.79 for the Form A and 0.55 for the Form B, generally indicating acceptable values. In another study,^18^ the Cronbach’s alpha values were estimated to be .69 and .60 for the Form A and Form B, respectively. In order to analyze the data, descriptive statistics as well as one-way analysis of variance (ANOVA) and multivariate analysis of variance (MANOVA) were run with the SPSS software (version 21, Chicago, IL, USA).

## RESULTS

Eighty-five percent (n=40) of women who had undergone cosmetic surgery, 80% (n=32) of women seeking cosmetic surgery and 86% (n=39) of ordinary women had academic education. Moreover, the data revealed that the age range in 34% (n=16) of women undergoing cosmetic surgery, 46.3% (n=19) of women seeking cosmetic surgery and 46.7% (n=21) of ordinary women were between 21 and 40 years. Also, 55.3% (n=26) of women having cosmetic surgery, 43.9% (n=18) of women seeking cosmetic surgery and 46.7% (n=21) of ordinary women were 41 years of age and older. The age mean and standard deviation were 42.26±5.77; 39.58±6.63; and 40.91±5.6 among the participants in the surgery, applicant and control group, respectively. 

Table 1, based on variance analysis, shows that significant differences were observed among the subjects in the three groups of surgery, applicant and control in terms of all the marital satisfaction components except for the components of sexual relations, marriage, children, and religious orientation, and, also, the three groups were significantly different in terms of the total marital satisfaction score (*p*=0.002, F 2,147=6.424). 

**Table 1:** The differences among the subjects in the three groups of surgery, applicant and control in terms of all the marital satisfaction components

**Variable**	**Source**	**df**	**Mean Square**	**F**	**P value**
Personality issues	Between groups	2	4.718	6.589	<0.001
	Within groups	147	0.716		
	Total	149			
Communication	Between groups	2	2.655	3.664	0.02
	Within groups	147	0.724		
	Total	149			
Conflict resolution	Between groups	2	4.610	8.095	<0.001
	Within groups	147	0.570		
	Total	149			
Financial management	Between groups	2	2.419	3.778	0.02
	Within groups	147	0.640		
	Total	149			
Leisure activities	Between groups	2	3.862	5.667	<0.001
	Within groups	147	0.682		
	Total	149			
Sexual relationship	Between groups	2	2.096	1.769	0.17
	Within groups	147	1.184		
	Total	149			
Children and parenting	Between groups	2	0.212	0.483	0.61
	Within groups	147	0.438		
	Total	149			
Family and friends	Between groups	2	2.094	4.449	0.01
	Within groups	147	0.471		
	Total	149			
Religious orientation	Between groups	2	0.904	1.604	0.20
	Within groups	147	0.563		
	Total	149			
Overall marital satisfaction	Between groups	2	2.146	6.424	<0.001
	Within groups	147	0.334		
	Total	149			

Table 2 shows that the surgery group and the applicant group were significantly different from each other in terms of the adjusted marital satisfaction mean scores; this means that the subjects in the applicant group reported higher marital satisfaction. Moreover, the adjusted mean scores of the marital satisfaction components were significantly different in the applicant group, as compared with both the surgery and control group, Accordingly, the applicant group reported higher satisfaction than the surgery and control group in terms of the personality issues component, higher satisfaction than the control group in terms of the communication component, higher satisfaction than the surgery and control group in terms of the conflict resolution and leisure activities components, and higher satisfaction than the surgery group in terms of the financial management and family and friends components. 

**Table 2:** Results of comparison of pairs of adjusted means of the marital satisfaction components at the group level using Tukey’s test

**Components of marital satisfaction**	**Groups**	**P value**
Personality issues	Requesting for cosmetic surgery VS who had surgery	0.01
	non-applicants VS who had surgery	0.89
	non-applicants VS requesting for cosmetic surgery	<0.001
Communication	Requesting for cosmetic surgery VS who had surgery	0.11
	non-applicants VS who had surgery	0.90
	non-applicants VS requesting for cosmetic surgery	0.03
Conflict resolution	Requesting for cosmetic surgery VS who had surgery	<0.001
	non-applicants VS who had surgery	0.85
	non-applicants VS requesting for cosmetic surgery	<0.001
Financial management	Requesting for cosmetic surgery VS who had surgery	0.02
	non-applicants VS who had surgery	0.53
	non-applicants VS requesting for cosmetic surgery	0.18
Leisure activities	Requesting for cosmetic surgery VS who had surgery	0.03
	non-applicants VS who had surgery	0.90
	non-applicants VS requesting for cosmetic surgery	<0.001
Sexual relationship	Requesting for cosmetic surgery VS who had surgery	0.81
	non-applicants VS who had surgery	0.48
	non-applicants VS requesting for cosmetic surgery	0.15
Children and parenting	Requesting for cosmetic surgery VS who had surgery	0.60
	non-applicants VS who had surgery	0.76
	non-applicants VS requesting for cosmetic surgery	0.95
Family and friends	Requesting for cosmetic surgery VS who had surgery	0.01
	non-applicants VS who had surgery	0.48
	non-applicants VS requesting for cosmetic surgery	0.13
Religious orientation	Requesting for cosmetic surgery VS who had surgery	0.17
	non-applicants VS who had surgery	0.61
	non-applicants VS requesting for cosmetic surgery	0.63
Overall marital satisfaction	Requesting for cosmetic surgery VS who had surgery	<0.001
	non-applicants VS who had surgery	0.98
	non-applicants VS requesting for cosmetic surgery	<0.001

According to the variance analysis results shown in Table 3, significant differences were observed among the subjects in the three groups of surgery, applicant and control in terms of ideal self-concept and overall self-concept score; however, the three groups were observed to be similar regarding the actual self-concept score. Table 4 shows that no significant difference was observed in the adjusted real self-concept mean scores between the three groups of surgery, applicant and control. The results with regard to ideal self-concept, however, indicated a meaningful difference in the surgery group, as compared to the control. Moreover, regarding the final self-concept score, resulting from the difference between real and ideal self-concept, the control group demonstrated higher positive self-concept compared to the other groups. Here, lower self-concept score indicates a positive self-concept. This means that the difference between the real and ideal self-concept in the normal group is less than that in the other two groups. 

**Table 3:** Results of ANOVA on the mean score of actual self-concept, ideal self-concept and self-concept

**Variable**	**Source**	**df**	**Mean Square**	**F**	**P value**
Real self-concept	Between groups	2	74.228	0.713	0.49
	Within groups	147	104.078		
	Total	149			
Ideal self-concept	Between groups	2	453.152	4.089	0.01
	Within groups	147	110.811		
	Total	149			
Self-concept	Between groups	2	597.420	9.147	0.001
	Within groups	147	65.310		
	Total	149			

**Table 4:** Results of comparison of pairs of adjusted means of actual self-concept, ideal self-concept and self-concept at the group level using Tukey’s test

**Components of marital satisfaction**	**Groups**	**P value**
Real self-concept	Requesting for cosmetic surgery VS who had surgery	0.58
	non-applicants VS who had surgery	1.0
	non-applicants VS requesting for cosmetic surgery	0.53
Ideal self-concept	Requesting for cosmetic surgery VS who had surgery	0.50
	non-applicants VS who had surgery	0.01
	non-applicants VS requesting for cosmetic Surgery	0.19
Self-concept	Requesting for cosmetic surgery VS who had surgery	0.97
	non-applicants VS who had surgery	<0.001
	non-applicants VS Applying for cosmetic surgery	<0.001

## DISCUSSION

According to the Table 1, it was shown that marital satisfaction level is different among people undergoing cosmetic surgery, surgery applicants and ordinary people. Based on Table 2 information, it was found that cosmetic surgery and to be more precise, expectation of positive outcomes after cosmetic surgery (the applicant group) had a positive impact, although perhaps temporarily, on the marital satisfaction components. Therefore, we can conclude that the first subsidiary hypothesis of this study is confirmed. 

As Table 3 shows, various levels of self-concept can be demonstrated in people undergoing cosmetic surgery, surgery applicants and ordinary people. Findings from this study revealed that the groups were significantly different in terms of marital satisfaction; accordingly, marital satisfaction was higher in the applicant and surgery group than in the control group. The results obtained in this study are indicative of the impact of cosmetic surgery on marital satisfaction. In addition, according to the results obtained by the examination of the marital satisfaction components, the subjects in the three groups of surgery, applicant and control were significantly different in terms of all the marital satisfaction components except for the components of sexual relations, marriage, children, and religious orientation. 

This means that the subjects in the applicant group reported higher satisfaction in most of the components. Accordingly, the applicant group reported higher satisfaction than the surgery and control group in terms of the personality issues, conflict resolution and leisure activities components, higher satisfaction than the control group in terms of the communication component, and higher satisfaction than the surgery group in terms of the financial management and family and friends’ components. 

These findings are consistent with the results of another study^19^ on the effects of cosmetic surgery on marital satisfaction. The participating women in their study regarded the cosmetic surgery-induced beauty as contributing to greater self-confidence, greater success in married life and higher social status and prestige, as well as a factor to reach power in the family; these researchers argue that, according to Giddens in this sense, human body turns into a place to create and fulfill people’s wishes and aspirations.

Also, the results of this study are in line with the results of other researchers^20^ on motivational factors influencing demand for breast surgery showing that the subjects reported a greater sense of femininity, higher attractiveness, less feeling of shame in the presence of men, improved sex life, and facilitated finding of partners as important motivational factors considered in demand for cosmetic surgery. In this context, the findings of this study are consistent with the results of another study too^21^ assessing the relationship between applying for cosmetic surgery and perfectionism, appearance schemas, satisfaction with romantic styles and relations. They suggested that the demand for cosmetic surgery can be predicted based on the dimension perfectionism, appearance schemas and relation satisfaction.

The findings of this research study are in a same line with the findings emphasizing the importance of psychosocial factors^6^^,^^22^^,^^23^ and psychological variables affecting the demand for cosmetic surgery. The results of this study are in similar to the results of research carried out before,^24^ showing that physical attractiveness plays an important role in romantic relations even after initiating a relation. According to the results, it may be argued that planning for cosmetic surgery and, more precisely, the applicants’ expectations for the positive outcome of the cosmetic surgery can have a positive impact on the marital satisfaction components.

The findings showed that a statistically significant difference was observed between the surgery and applicants group and control group, which means that the subjects in these two groups received higher self-concept scores. Since the higher self-concept scores reflect the weaker self-concept, the control group had higher levels of self-concept. The results of this study also showed no significant difference in real self-concept scores of the three groups including surgery applicants, surgery group and control group. Regarding the ideal self-concept, the results showed that the ideal self-concept was higher in surgery group than the control group and the difference was statistically significant. 

According to the results, it can be noted that one of the main reasons explaining individuals’ tendency towards cosmetic surgery may be the applicant’s low self-concept. Therefore, the second hypothesis of this study is confirmed. Similarly, it was shown that people who had cosmetic surgery possessed a body image weaker than those who had no interest in cosmetic surgeries. More specifically, overweight individuals were more interested in liposuction cosmetic surgery and had a body image weaker than the others.^25^

In this regard, the results of this study are in line with the findings of other authors^22^ found a significant relationship between gender and age with acceptance of cosmetic surgery. This relationship can be explained by mediating variables including personality, self-esteem, conformity and physical attractiveness. These results provide a preliminary model integrating personality traits and individual differences in predicting the acceptance of cosmetic surgery.

On the other hand, the results of this study indicating the lack of cosmetic surgery effect on self-concept in women who have had cosmetic surgery are not in a same vein with the findings obtained before.^26^ In their research, they investigated the effects of plastic surgery on body image, self-esteem and psychological problems prior to and 6 months after performing plastic surgery and found significant improvement in postoperative body image (satisfaction with physical appearance) of the experimental group. In this regard, the results of some research suggest that individuals report a better body image after cosmetic surgery procedures.^26^

So it can be concluded that the people who have cosmetic surgery as well as cosmetic surgery applicants in comparison with ordinary people have lower levels of self-concept. Further, as a majority of research studies conducted in this area confirm, self-concept is an important variable predicting the demand for a variety of cosmetic surgeries. These finding have implications for a lot of cosmetic surgery psychiatrists, psychologists, counselors and specialists. Undoubtedly, the identification of psychological factors related to the demand for cosmetic surgery procedures prevents the possible negative consequences of actions induced by psychological factors but not medical necessity. Therefore, expectation for postoperative positive outcome is an important factor affecting high level of marital satisfaction in surgery applicants. Furthermore, the component self-concept is also a significant predictor of having or applying for cosmetic surgery.

## CONFLICT OF INTEREST

The authors declare no conflict of interest.
